# The Associations Between Sleep Architecture and Metabolic Parameters in Patients With Obstructive Sleep Apnea: A Hospital-Based Cohort Study

**DOI:** 10.3389/fneur.2021.606031

**Published:** 2021-02-10

**Authors:** Nana Feng, Jundong Yang, Huajun Xu, Chujun Zhang, Fan Wang, Xiaolin Wu, Meizhen Gu

**Affiliations:** ^1^Department of Respiratory and Critical Medicine, Shanghai Eighth People's Hospital Affiliated to Jiangsu University, Shanghai, China; ^2^Department of Medicine, Jiangsu University, Zhenjiang, China; ^3^Department of Otolaryngology Head and Neck Surgery and Center of Sleep Medicine, Shanghai Jiao Tong University Affiliated Sixth People's Hospital, Shanghai, China; ^4^Otolaryngological Institute of Shanghai Jiao Tong University, Shanghai, China; ^5^Shanghai Key Laboratory of Sleep Disordered Breathing, Shanghai, China; ^6^Central Laboratory of Shanghai Eighth People's Hospital Affiliated to Jiangsu University, Shanghai, China; ^7^Department of Otolaryngology-Head & Neck Surgery, Shanghai Children's Hospital, Shanghai Jiao Tong University, Shanghai, China

**Keywords:** obstructive sleep apnea, metabolism, sleep architecture, polysomnography, sleep apnea

## Abstract

**Background and Objectives:** The associations between objective sleep architecture and metabolic parameters have been rarely studied in patients with obstructive sleep apnea (OSA). Here, we evaluated the associations between objective sleep measures derived *via* polysomnography (PSG) and metabolic parameters.

**Methods:** A total of 2,308 subjects with suspected OSA were included. We measured common metabolic parameters such as body mass index (BMI) and glucose, insulin, blood pressure, total cholesterol (TC), triglyceride (TG), high-density lipoprotein cholesterol (HDL-C), and low-density lipoprotein cholesterol (LDL-C) levels. All subjects underwent full-night PSG. PSG sleep parameters included total sleep time (TST), time spent in slow-wave sleep (SWS) and rapid eye movement (REM) sleep, sleep efficiency, and the microarousal index (MAI).

**Results:** The TST correlated with the BMI, glucose level, and systolic blood pressure. The SWS/TST ratio correlated with BMI and glucose, TC, and TG levels. The REM/TST ratio correlated with BMI, glucose, insulin, and TG levels, and diastolic blood pressure. We found significant relationships between sleep efficiency and BMI, glucose levels, and TG levels. The MAI was significantly correlated with all metabolic parameters. After adjustment for age, gender, smoking status, alcohol use, apnea hypopnea index, and oxygen desaturation index (ODI), multiple linear regression analysis showed that the MAI was independently associated with glucose level, TC, HDL, and LDL. REM/TST ratio was positively associated with diastolic blood pressure but negatively associated with glucose metabolism.

**Conclusions:** Though some independent correlation between sleep and metabolic parameters was confirmed, only weak associations were observed, suggesting a clinically negligible influence of sleep structure. Further prospective studies are warranted to confirm our findings.

## Introduction

Cardiovascular disease (CVD) is a leading cause of death worldwide. CVD prevalence is rising with increasing tobacco and alcohol use, physical inactivity, and consumption of high-fat/sugar diets (1). Obstructive sleep apnea (OSA) is a risk factor for CVD (2). OSA is commonly associated with common cardiovascular risk factors such as certain components of metabolic syndrome (MS) including dyslipidemia, insulin resistance, hypertension, diabetes, and obesity (3–6). Both subjective and objective sleep disturbances increase CVD risk. Between 6 and 8 h of sleep per day reduces the risk of major cardiovascular events (7). Weak associations have been reported among excessive daytime sleepiness, CVD, and coronary heart disease risk (8). The apnea–hypopnea index (AHI) and oxygen desaturation index (ODI), derived *via* polysomnography (PSG), were independently associated with cardiovascular risk factors including mean oxygen saturation (9). However, the contributions of other OSA-related sleep characteristics (quantitative measures of sleep disturbance) to common CVD risk (i.e., metabolic parameters) have not been well-studied. In particular, few studies have explored the relationships of total sleep time (TST), time spent in slow-wave sleep (SWS) and rapid eye movement (REM) sleep, sleep efficiency, and the microarousal index (MAI) with metabolic parameters. Thus, this study tested for independent associations between these sleep parameters (based on PSG) and the metabolic risk factors of obesity, high glucose and insulin levels, and a disturbed lipid profile in patients with OSA. We hypothesized that subjects with poor sleep characteristics were more likely to have detrimental metabolic statuses.

## Methods

### Population Sampling

This observational study consecutively enrolled adults who visited the sleep center of Shanghai Jiao Tong University Affiliated Sixth People's Hospital from January 2010 to December 2015. The study was approved by the Ethics Committee of our hospital [2019-KY-050(K)]. Informed consent was obtained from all participants, who completed questionnaires on medical history, smoking status, alcohol use, and subjective sleep quality. The subjects were classified as smokers (ex- or current) or never-smokers and as current alcohol drinkers or nondrinkers. The exclusion criteria were as follows: (1) aged <18 years; (2) previous upper airway surgery, use of an oral appliance, or continuous positive airway pressure treatment; (3) severe chronic systemic disease (i.e., hepatic, pulmonary, and cardiac failure); (4) another sleep disorder (severe insomnia, restless leg syndrome, or narcolepsy) or a mental condition; (5) current use of antipsychotics; (6) use of medications to treat hypertension, diabetes mellitus, or hyperlipidemia; and (7) missing data. Ultimately, 2,308 participants were included in the analysis.

### Metabolic Parameters

Height and weight were measured as described previously (10). Blood pressure was measured twice using the left arm after a 15-min rest period in the evening at the time of the study and blood pressure was taken at the same time (8:00 pm) for all participants, and the mean value was calculated. Fasting venous blood was collected from each participant at 07:00 for all participants. Fasting venous blood was collected for analyses of insulin and glucose levels and the lipid profile [total cholesterol (TC), triglycerides (TGs), high-density lipoprotein cholesterol (HDL-C), and low-density lipoprotein cholesterol (LDL-C)] in the laboratory of our hospital by the same procedures, i.e., serum lipid and fasting serum glucose levels were measured in the hospital laboratory using an autoanalyzer (H-7600; Hitachi, Tokyo, Japan). The body mass index (BMI) was calculated using height and weight data. MS was defined as suggested by the Adult Treatment Panel Report III based on the presence of at least three of the following: (1) abdominal obesity [waist circumference (WC) >102 and 88 cm in males and females, respectively]; (2) elevated TG level [≥1.7 mmol/L (>150 mg/dl)]; (3) low HDL-C level [ <1.03 mmol/L (40 mg/dl) and <1.20 mmol/L (50 mg/dl) in males and females, respectively]; (4) elevated blood pressure (≥130/≥85 mmHg) or a history of hypertension; and (5) an elevated fasting blood glucose level (≥5.6 mmol/L) or any history of type 2 diabetes mellitus (11).

### Polysomnography

All participants underwent full-night PSG (Alice 4 or Alice 5; Respironics, Pittsburgh, PA, USA). The data were recorded using 18 channels: six for electroencephalography (EEG), two for electrooculography (EOG), two for surface (chin) electromyography (EMG), three for electrocardiography (ECG), and one each for oronasal temperature, nasal pressure, thoracic and abdominal belt, body position, and oxygen saturation. Polysomnographic variables were scored manually by skilled technicians according to the 2012 guidelines of the American Academy of Sleep Medicine (AASM) (12); if the PSG were recorded before 2012, they were reanalyzed according to the same 2012 AASM criteria. Apnea was defined as at least 90% reduction in airflow for at least 10 s; hypopnea was defined as at least 50% reduction in airflow for at least 10 s or longer and accompanied by at least 3% reduction in oxygen saturation or arousal, as described previously (5). The AHI was given by the average number of apneas/hypopneas per hour of sleep. The ODI was given by the number of times that oxygen saturation fell by more than 3% per hour of sleep. The MAI was given by the mean number of arousals per hour of sleep. The TST was recorded as the total number of minutes of any form of sleep, from onset to morning awakening. Sleep efficiency was given by the ratio of TST to the time spent in bed. SWS (stage N3) and REM sleep durations were also recorded.

### Statistical Analysis

All statistical tests were performed using SPSS software (ver. 22.0; IBM SPSS Statistics, IBM Corp., Armonk, NY, USA), and a two-sided *p*-value <0.05 was considered significant. The raw data were subjected to Kolmogorov–Smirnov testing. Non-normally distributed data were normalized *via* log transformation. Continuous variables are shown as mean ± standard deviation and categorical variables as percentages. Student's *t*-test or one-way analysis of variance (ANOVA) was used to test for differences between the groups in univariate variables. Spearman correlation analysis was used as appropriate to examine bivariate associations between sleep architecture and metabolic variables. Multiple linear regression models were used to identify independent relationships between sleep parameters and metabolic variables after controlling for age, sex, smoking status, and alcohol consumption or with additional AHI and ODI.

## Results

### Baseline Characteristics

A total of 2,308 subjects who were primary snorers were included in the final analysis. Their baseline anthropometric, metabolic, and polysomnographic data are listed in Table 1. We assigned the participants to two groups according to the AHI; one group had none-to-mild OSA (AHI <15/h) (*n* = 1,021) and the other had moderate-to-severe OSA (AHI ≥15/h) (*n* = 1,287). Compared to those with none-to-mild OSA, subjects with moderate-to-severe OSA were older (41.20 vs. 38.84 years, *p* < 0.001) and had higher BMI (27.05 vs. 24.10 kg/m^2^, *p* < 0.001), systolic blood pressure (SBP) (125.66 vs. 120.41 mmHg, *p* < 0.001), and diastolic blood pressure (DBP) (80.88 vs. 77.12, *p* < 0.001) values, as well as higher glucose (5.45 vs. 5.05 mmol/L, *p* < 0.001), insulin (13.33 vs. 9.01 μU/mL, *p* < 0.001), TC (4.86 vs. 4.50 mmol/L, *p* < 0.001), TG (2.11 vs. 1.42 mmol/L, *p* < 0.001), and LDL (3.08 vs. 2.78 mmol/L, *p* < 0.001) levels. In terms of objective sleep parameters, patients with moderate-to-severe OSA spent less time in SWS (11.49 vs. 14.92%, *p* < 0.001) and REM sleep (8.88 vs. 10.38%, *p* < 0.001) and had higher sleep efficiency (0.82 vs. 0.78, *p* < 0.001) and MAI values (31.38 vs. 17.65/h, *p* < 0.001).

**Table 1:** Basic characteristics of all participants.

**Characteristic**	**All (*n* = 2,308)**	**AHI <15/h(*n* = 1,021)**	**AHI ≥15/h (*n* = 1,287)**	***p*-value**
**Demographics**				
Age, years	40.16 ± 11.18	38.84 ± 11.58	41.20 ± 10.74	<0.001
Male (%)	79.5	66.8	89.7	<0.001
BMI, kg/m^2^	25.74 ± 3.77	24.10 ± 3.31	27.05 ± 3.61	<0.001
Smokers, *n* (%)	773 (33.5)	237 (23.2)	536 (41.6)	<0.001
Alcohol consumption, *n* (%)	1,028 (44.5)	373 (36.5)	655 (50.9)	<0.001
**Biochemical variables**				
Glucose (mmol/L)	5.27 ± 0.99	5.05 ± 0.81	5.45 ± 1.08	<0.001
Insulin (μU/mL)	11.41 ± 7.95	9.01 ± 5.61	13.33 ± 8.96	<0.001
SBP, mmHg	123.33 ± 14.69	120.41 ± 13.60	125.66 ± 15.11	<0.001
DBP, mmHg	79.22 ± 10.94	77.12 ± 10.02	80.88 ± 11.34	<0.001
TC (mmol/L)	4.70 ± 0.97	4.50 ± 0.99	4.86 ± 0.93	<0.001
TG (mmol/L)	1.80 ± 1.48	1.42 ± 1.02	2.11 ± 1.71	<0.001
HDL (mmol/L)	1.13 ± 2.57	1.14 ± 0.27	1.13 ± 3.43	0.915
LDL (mmol/L)	2.94 ± 0.8	2.78 ± 0.80	3.08 ± 0.78	<0.001
**Polysomnography**				
Total sleep time, min	373.65 ± 73.08	363.50 ± 76.54	381.63 ± 69.18	<0.001
SWS, % of TST	13.0 ± 10.7	14.92 ± 10.67	11.49 ± 10.47	<0.001
REM, % of TST	9.54 ± 6.86	10.38 ± 7.11	8.88 ± 6.59	<0.001
Sleep efficiency, %	0.80 ± 0.16	0.78 ± 0.17	0.82 ± 0.16	<0.001
MAI	25.34 ± 19.67	17.65 ± 14.09	31.38 ± 21.26	<0.001
AHI	28.54 ± 26.72	4.94 ± 4.32	47.22 ± 21.78	<0.001
ODI	29.36 ± 28.20	7.16 ± 13.21	46.94 ± 24.25	<0.001

Data are presented as numbers (percentages) or mean ± SD.

Differences in baseline characteristics between patients with AHI <15/h and AHI ≥15/h were explored using the independent t-test or chi-square test.

*AHI, apnea–hypopnea index; BMI, body mass index; SBP, systolic blood pressure; DBP, diastolic blood pressure; TC, total cholesterol; TG, triglyceride; HDL-C, high-density lipoprotein cholesterol; LDL-C, low-density lipoprotein cholesterol; SWS, slow-wave sleep; TST, total sleep time; REM, rapid eye movement; ODI, oxygen desaturation index; MAI, microarousal index*.

### Relationships Between Sleep Parameters and Metabolic Parameters

The TST correlated with BMI, glucose level, and SBP. The SWS/TST ratio correlated with BMI and glucose, TC, and TG levels. The REM/TST ratio correlated with BMI glucose, insulin and TG levels, and DBP. Sleep efficiency was significantly associated with BMI and glucose and TG levels. The MAI correlated significantly with all metabolic parameters (Table 2).

**Table 2:** Bivariate correlations between sleep parameters and metabolic variables in all subjects.

	**BMI (kg/m^**2**^)**	**Glucose** **(mmol/L)**	**Insulin** **(μU/mL)**	**SBP** **(mmHg)**	**DBP** **(mmHg)**	**TC** **(mmol/L)**	**TG** **(mmol/L)**	**HDL** **(mmol/L)**	**LDL** **(mmol/L)**
Total sleep time, min	0.093^**^	0.075^**^	0.022	0.042^*^	0.040	0.003	0.031	0.006	−0.016
SWS, % of TST	−0.119^**^	−0.086^**^	−0.005	−0.034	−0.011	−0.058^**^	−0.056^**^	0.027	−0.029
REM, % of TST	−0.099^**^	−0.075^**^	−0.053^*^	0.034	0.058^**^	−0.036	−0.064^**^	0.014	0.008
Sleep efficiency, %	0.105^**^	0.089^**^	0.003	0.006	0.002	0.018	0.042^*^	−0.005	0.020
MAI	0.230^**^	0.145^**^	0.225^**^	0.090^**^	0.137^**^	0.134^**^	0.145^**^	0.050^*^	0.182^**^

*BMI, body mass index; SBP, systolic blood pressure; DBP, diastolic blood pressure; TC, total cholesterol; TG, triglyceride; HDL-C, high-density lipoprotein cholesterol; LDL-C, low-density lipoprotein cholesterol; SWS, slow-wave sleep; TST, total sleep time; REM, rapid eye movement; MAI, microarousal index*.

*All numbers represent correlation coefficients*.

Spearman correlation analysis:

** significant at the 0.01 level;

* *significant at the 0.05 level*.

To minimize the effect of OSA, we excluded subjects with a high AHI (≥15/h). Sensitivity analysis revealed significant correlations between the TST and HDL and LDL levels; between the SWS/TST ratio and glucose level; between the REM/TST ratio and insulin and TG levels, SBP, and DBP; between sleep efficiency and the HDL level; and between the MAI and TC and LDL levels (Table 3).

**Table 3:** Bivariate correlations between sleep parameters and metabolic variables in subjects without moderate-to-severe OSA.

	**BMI (kg/m^**2**^)**	**Glucose (mmol/L)**	**Insulin (μU/ml)**	**SBP (mmHg)**	**DBP** **(mmHg)**	**TC (mmol/L)**	**TG** **(mmol/L)**	**HDL (mmol/L)**	**LDL** **(mmol/L)**
Total sleep time, min	0.029	0.046	0.036	0.006	0.001	−0.048	0.019	−0.096^**^	−0.074^*^
SWS, % of TST	−0.048	−0.069^*^	0.052	−0.007	0.024	−0.016	−0.032	0.014	0.023
REM, % of TST	−0.034	−0.040	−0.082^**^	0.068^*^	0.071^*^	−0.006	−0.067^*^	0.021	0.012
Sleep efficiency, %	0.026	0.045	0.006	−0.012	−0.032	−0.037	0.015	−0.065^*^	−0.050
MAI	0.041	0.010	0.051	−0.020	−0.017	0.078^*^	0.032	0.029	0.134^**^

OSA, obstructive sleep apnea; BMI, body mass index; SBP, systolic blood pressure; DBP, diastolic blood pressure; TC, total cholesterol; TG, triglyceride; HDL-C, high-density lipoprotein cholesterol; LDL-C, low-density lipoprotein cholesterol; SWS, slow-wave sleep; TST, total sleep time; REM, rapid eye movement; MAI, microarousal index.

** significant at the 0.01 level.

* *significant at the 0.05 level*.

We performed multiple linear regression analyses to identify independent relationships between sleep parameters and metabolic variables after adjusting for multiple confounding factors, including age, sex, smoking status, and alcohol use (model 1). The TST, SWS/TST ratio, and MAI were independent predictors of BMI. Also, sleep efficiency and the MAI were independent predictors of glucose level. The MAI was an independent predictor of insulin level, blood pressure, and the lipid profile (Table 4). REM/TST ratio was negatively associated with BMI, glucose level, insulin level, and TG level. The results were stable when we excluded subjects with a high AHI (≥15/h) (Table 5). After adjusting for variables in model 1 and additional AHI and ODI (model 2), sleep efficiency and the MAI were independent predictors of glucose level. The MAI was an independent predictor of TC, HDL, and LDL level. The REM/TST ratio was negatively associated with BMI, glucose level, and TG level (Table 4). Similarly, the results were also stable when we excluded moderate to severe OSA (Table 5).

**Table 4:** Multiple linear regression analysis of the effects of sleep parameters on metabolic variables in the overall population.

	**Model 1**				**Model 2**			
**Outcome**	**Predictor**	***β***	**Standard error**	***p*-value**	**Predictor**	***β***	**Standard error**	***p*-value**
BMI (kg/m^2^)	MAI	0.037	0.004	<0.001	REM/TST (%)	−0.025	0.011	0.016
	SWS/TST (%)	−0.030	0.007	<0.001				
	TST	0.004	0.001	<0.001				
	REM/TST (%)	−0.042	0.011	<0.001				
Glucose (mmol/L)	MAI	0.007	0.001	<0.001	MAI	0.002	0.001	0.036
	SE (%)	0.406	0.125	0.001	SE (%)	0.323	0.125	0.01
	REM/TST (%)	−0.007	0.003	0.017	REM/TST (%)	−0.006	0.003	0.048
Insulin (μU/mL)	MAI	0.086	0.008	<0.001	(-)	(-)	(-)	(-)
	REM/TST (%)	−0.076	0.023	0.001				
SBP (mmHg)	MAI	0.051	0.016	<0.001	(-)	(-)	(-)	(-)
DBP (mmHg)	MAI	0.062	0.012	<0.001	REM/TST (%)	0.132	0.033	<0.001
	REM/TST (%)	0.103	0.033	0.002				
TC (mmol/L)	MAI	0.006	0.001	<0.001	MAI	0.003	0.001	0.004
TG (mmol/L)	MAI	0.009	0.002	<0.001	REM/TST (%)	−0.009	0.004	0.043
	REM/TST (%)	−0.012	0.004	0.006				
HDL (mmol/L)	MAI	0.008	0.003	0.005	MAI	0.011	0.003	<0.001
LDL (mmol/L)	MAI	0.007	0.001	<0.001	MAI	0.005	0.001	<0.001

Model 1 was adjusted for age, sex, smoking status, and alcohol use; model 2 was adjusted for variables in model 1 and additional AHI and ODI.

*BMI, body mass index; SBP, systolic blood pressure; DBP, diastolic blood pressure; TC, total cholesterol; TG, triglyceride; HDL-C, high-density lipoprotein cholesterol; LDL-C, low-density lipoprotein cholesterol; SE, sleep efficiency; TST, total sleep time; REM, rapid eye movement; MAI, microarousal index*.

**Table 5:** Multiple linear regression analysis of the effects of sleep parameters on metabolic variables in subjects without OSA.

	**Model 1**				**Model 2**			
**Outcome**	**Predictor**	***β***	**Standard error**	***p*-value**	**Predictor**	***β***	**Standard error**	***p*-value**
BMI (kg/m^2^)	(-)	(-)	(-)	(-)	(-)	(-)	(-)	(-)
Glucose (mmol/L)	(-)	(-)	(-)	(-)	(-)	(-)	(-)	(-)
Insulin (μU/mL)	REM/TST (%)	−0.074	0.025	0.003	REM/TST (%)	−0.071	0.025	0.005
	SWS/TST (%)	0.035	0.017	0.038	SWS/TST (%)	0.035	0.017	0.038
SBP (mmHg)	REM/TST (%)	0.156	0.059	0.008	REM/TST (%)	0.164	0.059	0.005
DBP (mmHg)	REM/TST (%)	0.109	0.044	0.014	REM/TST (%)	0.110	0.044	0.013
TC (mmol/L)	MAI	0.005	0.002	0.012	MAI	0.005	0.002	0.036
TG (mmol/L)	(-)	(-)	(-)	(-)	(-)	(-)	(-)	(-)
HDL (mmol/L)	TST	0.01	0.01	<0.001	TST	0.000	0.000	0.001
LDL (mmol/L)	MAI	0.007	0.002	<0.001	MAI	0.006	0.002	0.001

Model 1 was adjusted for age, sex, smoking status, and alcohol use; model 2 was adjusted for variables in model 1 and additional AHI and ODI.

*OSA, obstructive sleep apnea; BMI, body mass index; SBP, systolic blood pressure; DBP, diastolic blood pressure; TC, total cholesterol; TG, triglyceride; HDL-C, high-density lipoprotein cholesterol; LDL-C, low-density lipoprotein cholesterol; TST, total sleep time; REM, rapid eye movement; MAI, microarousal index*.

As we can see, even when excluding patients with AHI ≥15 from the multivariate analysis, though some independent correlation between sleep and metabolic parameters was confirmed, only weak associations were observed, suggesting a clinically negligible influence of sleep structure.

## Discussion

To the best of our knowledge, few studies have explored the association between sleep architecture and metabolic status. We enrolled a relatively large number of subjects and found that many metabolic variables were affected by sleep parameters. A higher proportion of REM sleep was an independent risk factor for elevated blood pressure, and a higher MAI was an independent risk factor for suboptimal lipid metabolism. After controlling for OSA, these findings remained significant. However, though some independent correlation between sleep and metabolic parameters was confirmed, only weak associations were observed, suggesting a clinically negligible influence of sleep structure.

OSA is commonly accompanied by disturbed sleep architecture, including a reduced proportion of SWS sleep and lower sleep efficiency in children with a common phenotype of OSA (13). In adults with OSA, regardless of sleep duration, alterations in sleep architecture prevent adequate rest (14). We also found shorter SWS and REM sleep durations and a higher MAI in OSA patients. A previous study found that more SWS, increased sleep efficiency, and a greater TST improved glucose and insulin homeostasis in overweight/obese children and adolescents (15). In such adolescents, insufficient/excessive sleep was associated with hyperglycemia, and a decreased SWS was linked to a decrease in insulin level (16). However, it remains unclear how sleep architecture affects the metabolic status of adults.

During REM sleep, autonomic status is unstable, reflected in fluctuations in the parasympathetic and sympathetic nervous systems. As cortical desynchronization develops during REM sleep, the respiratory and circulatory systems become increasingly unstable (17), which will affect the blood pressure. We found that a longer REM sleep duration was an independent risk factor for higher blood pressure. Persistently high levels of sympathetic activity during REM sleep may explain the increases in blood pressure. Previous studies showed that AHI during REM sleep was independently associated with hypertension, insulin resistance, MS, and diabetes (18–21). The increased numbers of apneas and hypopneas during REM sleep may also explain why blood pressure increases. However, we found that longer REM sleep proportion was associated with lower insulin and glucose (Tables 4, 5). Few studies had explored the relationship between REM sleep proportion and glucose metabolism; further prospective or randomized controlled studies are warranted to confirm our findings.

Increased microarousal rate, one of the most important features of OSA, was shown to be independently associated with hyper-LDL cholesterolemia (22). We also found that the MAI was independently associated with lipid metabolism. There are several possible explanations for this: first, the MAI closely reflects sympathetic hyperactivity, which is associated with lipolysis. Second, microarousals may activate the hypothalamic–pituitary–adrenal (HPA) axis, further elevating the levels of cortisol and other hormones and thus triggering lipolysis (23). Third, microarousals are associated with systemic inflammation, which also plays an important role in lipid metabolism (24).

We found that SWS sleep duration was independently associated with insulin level, similar to a previous study reporting that decreased SWS was associated with a dose-dependent increase in OSA-associated hypertension (25). SWS is “restorative” sleep; during SWS, the sympathetic tone is weakened, slow-wave activity is increased, and cortisol secretion is decreased. Sympathetic overactivity caused by a shorter SWS stimulates insulin secretion. Thus, improvements in sleep architecture improve metabolic status.

The strengths of our study included the relatively large sample size, adequate adjustment for potential confounders, and use of standard PSG to obtain objective measures of sleep architecture. However, several potential limitations should also be addressed. First, the cross-sectional design did not allow us to infer causality. Second, we performed PSG only once, in a laboratory environment, so there may have been a “first night effect” and the sleep architecture might have differed somewhat from normal. Third, although we enrolled residents of southeast China with similar lifestyles, we did not adjust for various factors that affect metabolic variables, including dietary components and physical activity level. Finally, this was a hospital-based study, so the findings cannot be generalized to the general population. In conclusion, whether high-quality sleep of adequate duration is required to maintain metabolic status and might reduce the risk of CVD in OSA is still uncertain. Further prospective studies are warranted to confirm our findings.

## Data Availability Statement

The raw data supporting the conclusions of this article will be made available by the authors, without undue reservation.

## Ethics Statement

The studies involving human participants were reviewed and approved by Ethics Committee of Shanghai Jiao Tong University Affiliated Sixth People's Hospital. The patients/participants provided their written informed consent to participate in this study. Written informed consent was obtained from the individual(s) for the publication of any potentially identifiable images or data included in this article.

## Author Contributions

The authors take responsibility and vouch for the accuracy and completeness of the data and analyses. XW and MG had full access to all of the data in the study and took responsibility for the integrity of the data and the accuracy of the data analysis. HX, XW, and MG: study design. NF, JY, HX, FW, and CZ: data collection. HX, CZ, and XW: statistical analysis. HX, NF, XW, and MG: manuscript draft. All authors have seen and approved the manuscript.

## Conflict of Interest

The authors declare that the research was conducted in the absence of any commercial or financial relationships that could be construed as a potential conflict of interest.

## Abbreviations

OSA: obstructive sleep apnea
PSG: polysomnography
CVD: cardiovascular disease
BMI: body mass index
SBP: systolic blood pressure
DBP: diastolic blood pressure
TC: total cholesterol
TG: triglyceride
HDL-C: high-density lipoprotein cholesterol
LDL-C: low-density lipoprotein cholesterol
AHI: apnea–hypopnea index
ODI: oxygen desaturation index
MAI: microarousal index
TST: total sleep time
SWS: slow-wave sleep
REM: rapid eye movement
CVD: cardiovascular disease.
